# Systemic Response of Antioxidants, Heat Shock Proteins, and Inflammatory Biomarkers to Short-Lasting Exercise Training in Healthy Male Subjects

**DOI:** 10.1155/2021/1938492

**Published:** 2021-11-22

**Authors:** Ivan Dimauro, Elisa Grazioli, Veronica Lisi, Flavia Guidotti, Cristina Fantini, Cristina Antinozzi, Paolo Sgrò, Ambra Antonioni, Luigi Di Luigi, Laura Capranica, Daniela Caporossi

**Affiliations:** ^1^Unit of Biology and Genetics of Movement, Department of Movement, Human and Health Sciences, University of Rome Foro Italico, Piazza Lauro de Bosis 15, 00135 Rome, Italy; ^2^Sport Performance Laboratory, Department of Movement, Human and Health Sciences, University of Rome Foro Italico, Piazza Lauro de Bosis 15, 00135 Rome, Italy; ^3^Endocrinology Unit, Department of Movement, Human and Health Sciences, University of Rome Foro Italico, Piazza Lauro de Bosis 15, 00135 Rome, Italy

## Abstract

Regular physical activity can enhance immune function and effectively prevents the spread of the cytokine response, thus reducing systemic low-grade inflammation and improving various immune markers. Moreover, regular exercise maintains redox homeostasis in skeletal muscle and other tissues, including immune cells, but the interconnection between the anti-inflammatory effects of exercise with the redox status of immune cells is still poorly understood. With the aim to verify the overall beneficial effect of regular training on the immune system, we have examined the acute and short-term effect of a 5-day exercise program on the modulation of protein and lipid oxidation, antioxidants (i.e., superoxide dismutase-1 (SOD1) and superoxide dismutase-2 (SOD2), glutathione peroxide 1 (GPx1), thioredoxin reductase-1 (TrxR1), and catalase (CAT)), and heat shock protein expression (i.e., heat shock protein-70 (HSP70) and heat shock protein-27 (HSP27)), at both mRNA and protein levels, as well as the activation of the nuclear factor kappa light chain enhancer of activated B cells (NF*κ*B) in peripheral blood mononuclear cells (PBMCs). Moreover, plasmatic markers of oxidative stress, inflammation, and stress response (i.e., protein carbonyl content, interleukin-6 (IL6), interleukin-8 (IL8), interleukin-10 (IL10), interleukin-17E (IL17E), interleukin-17F (IL17F), interleukin-21 (IL21), interleukin-22 (IL22), and interleukin-23 (IL23)) were analyzed in active untrained young adult subjects. Even in the absence of an increased amount of protein or lipid oxidation, we confirmed a PBMC upregulation of SOD1 (1.26 ± 0.07 fold change, *p* < 0.05), HSP70 (1.59 ± 0.28 fold change, *p* < 0.05), and HSP27 gene expression (1.49 ± 0.09 fold change, *p* < 0.05) after 3 hours from the first bout of exercise, followed by an increase in proteins' amount at 24 hours (SOD1, 1.80 ± 0.34 fold change; HSP70, 3.40 ± 0.58 fold change; and HSP27, 1.81 ± 0.20 fold change, *p* < 0.05) and return to basal levels after the 5 days of aerobic training. Indeed, the posttraining basal levels of oxidized molecules in plasma and PBMCs were statistically lower than the pretraining levels (carbonyl content, 0.50 ± 0.05 fold change, *p* < 0.01), paralleled by a lower expression of SOD2, Gpx1, and TrxR1, at mRNA (SOD2, 0.63 ± 0.06; GPx1, 0.69 ± 0.07; and TrxR1, 0.69 ± 0.12 fold change, *p* < 0.05) and protein (TrxR1, 0.49 ± 0.11 fold change, *p* < 0.05) levels. These results verified the existence of an early phase of redox adaptation to physical exercise already achievable after 5 days of moderate, regular aerobic training. More interestingly, this phenomenon was paralleled by the degree of NF*κ*B activation in PBMCs and the decrease of plasmatic proinflammatory cytokines IL8, IL21, and IL22 in the posttraining period, suggesting an interconnected, short-term efficacy of aerobic exercise towards systemic oxidative stress and inflammation.

## 1. Introduction

There are substantial evidences that regular physical activity promotes health and healthy aging [1–3]. Indeed, regular exercise promotes multiple metabolic and immune benefits related to the decrease in the risk of various diseases, including, but not limited to, diabetes, cardiovascular disease, cancer, and Alzheimer's disease [4–6].

Chronic inflammatory status and imbalance of the redox homeostasis are hallmarks of most of the biological and pathological conditions benefitting from an active lifestyle or exercise intervention [7–10]. Moreover, the activation of the anti-inflammatory pathways and/or the improvement of antioxidant and stress response with reduction of oxidative damage might represent key cellular processes ultimately leading to a reduction in mortality risk [11–13]. There is a growing body of evidence indicating the positive effects of regular moderate exercise (65–85% of maximum heart rate (HRmax)) on immune competency in healthy young and/or elderly subjects [14, 15], and it is recognized that the altered redox state in immune cells is connected to different metabolic- and cardiovascular-related conditions [16]. Nevertheless, few evidences support the interaction between oxidative stress and proinflammatory cytokine production, and the interconnection between the anti-inflammatory effect of exercise and the redox status (i.e., oxidants and antioxidants) of immune cells has not been elucidated after a short-term lasting exercise protocol, yet [17, 18].

It is widely known that peripheral blood mononuclear cells (PBMCs) represent the front line of the human immune system as well as the most essential mediators of stress and inflammation by producing cytokines, chemokines, and growth factors that may lead to beneficial or even pathological effects on tissues [19–21]. As other cell types (e.g., muscle cells) [22], PBMCs synthesize inflammatory proteins and reactive oxygen species (ROS) that can cause damage and dysfunction to arteries and other tissues, and it has been postulated that a proinflammatory/oxidant gene expression profile in PBMC may contribute to increased risk of cardiovascular and other diseases [23]. On the other hand, data from our and other laboratories on trained subjects show that the adaptive response of PBMCs in terms of the modulation of antioxidants and stress-induced markers is linked to rapid and substantial changes in the gene expression pattern, which correlates with an improvement of other parameters of healthy status [4, 24–30].

To date, studies concerning the effects of exercise on immune cells have focused mainly on athletes, with the overall goals of ascertaining the extent of immune decline due to excessive exercise training [31, 32], or on elderly, to identify the factors responsible for the improvement of immune response by regular exercise training [1]. Given the positive immunological effects that a regular exercise of moderate intensity can induce also in healthy young/adults, such as the decrease of cytokine levels, the enhancement of vaccine response, and the prompt response to viral infections [33–38], we aimed to analyze the effect of a moderate and short-lasting exercise period (5 days) on the modulation of plasma pro- and anti-inflammatory cytokines (i.e., IL6, IL8, IL10, IL17E, IL17F, IL21, IL22, and IL23) of healthy male subjects with a medium fitness level (38 < VO_2max_ < 56 mL/kg/min), analyzing at the same time in the PBMCs the modulation of protein and lipid oxidation, antioxidant (i.e., SOD1, SOD2, GPx1, TrxR1, and CAT) and heat shock protein expression (i.e., HSP70 and HSP27), at both mRNA and protein levels, and the activation of the nuclear factor kappa light chain enhancer of activated B cells (NF*κ*B). Moreover, several functions of lymphocytes are strongly regulated by redox status, including activation, proliferation, and apoptosis [39]. Therefore, the analysis of these parameters is a priority in defining the redox status of the lymphocytes.

Our results highlight the existence of an early phase for the exercise-induced adaptation of the redox components in immune cells, also suggesting an interconnected, short-term efficacy of aerobic exercise towards improved systemic oxidative stress and inflammation. In particular, after the 5-day training, the levels of both oxidized molecules and antioxidant enzymes verified a better redox buffering capacity paralleled by low levels of NF*κ*B activation and a significant reduction of plasmatic proinflammatory cytokines IL8, IL21, and IL22.

## 2. Material and Methods

### 2.1. Study Design

A total of 10 healthy male subjects (26.6 ± 3.1 years) have been recruited for this study at the University of Rome “Foro Italico” (Table 1).

Specific eligibility criteria included male with an active lifestyle (<150 min/week recreational activity over the past 12 months) corresponding to a medium fitness level matched for age (36 < VO_2max_ < 47 mL/kg/min), age 20-30 years, no illness or ongoing medication, and no signs of cardiovascular, metabolic, and pulmonary disease, orthopaedic injury or joint disease, and neurological or immunologic disease.

All participants underwent a detailed medical history and physical examination and provided informed written consent approved by the Ethics Committee of the University of Rome “La Sapienza” (RIF.CE: 4521). Moreover, they completed a detailed eating habit diary in which were recorded all food and drinks consumed during the 3 consecutive days before beginning the training protocol.

### 2.2. Evaluation of Physical Activity Level and Exercise Protocol

As previously reported [30], before starting the acute endurance exercise protocol, each participant performed a physical fitness assessment to estimate VO_2max_. Briefly, the aerobic capacity was assessed using the Balke treadmill test [40], a continuous incremental test on a treadmill (Skillrun Treadmill, Technogym, Italy). The test began with a warm-up of 5 min, the slope was 0% and the speed 5.3 km/h; then, the operator increased the slope of 1° after 1 min and then every minute. The score of the test is the time spent walking or running on the treadmill till exhaustion, in minutes. In most cases, time spent on the treadmill should be between 9 and 15 min. It is possible to estimate the VO_2max_ score using the test time through the following formulas where the value “*T*” is the test time [VO_2max_ = 1.444 (*T*) + 14.99] [41]. The heart rate was monitored using a heart rate monitor chest strap and fatigue using the Borg Scale [42] in order to evaluate the right slope to reach the 70% of volunteer maximal heart rate. Instantly, 3 and 6 min after the end of the test, the lactate concentration was evaluated by a finger-stick lactate analyzer, in order to monitor the recovery of the subjects that is an important parameter to evaluate the fitness level; the longer the recovery time, the lower the training level of the subject [43]. Before the Balke test, each subject performed the Baecke questionnaire developed for evaluating a person's physical activity into three distinct dimensions/levels: work, sport, and leisure [44].

During the training period lasting 5 days, all subjects were involved in the same aerobic exercise. The session duration was 30 minutes each, using a treadmill set to 5.3 km/h speed. The % slope was individualized and adapted to each participant at 70% of the individual maximal heart rate.

### 2.3. Blood Lactate

**Figure 1:**
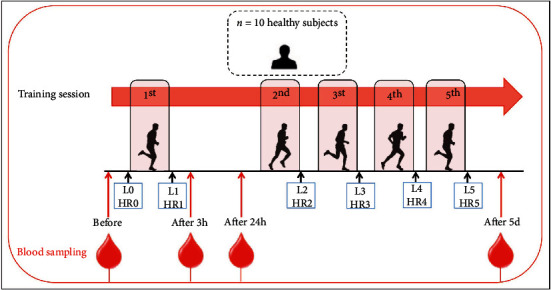
Schematic overview of blood sampling during exercise protocol. This scheme shows the blood sampling at rest (before), 3 h (after 3 h) and 24 h (after 24 h) after the 1^st^ training session, and 24 h after the 5^th^ training session (after 5 d). Moreover, the lactate level and heart rate are measured before (L0, HR0) and immediately after (L1-L5, HR1-HR5) each training session. L: lactate; HR: heart rate.

The lactate level was measured without interrupting the exercise session by taking capillary blood from a fingertip and analyzed with the ACUSPORT lactometer (Boehring Mannheim) at each training session as indicator of muscular adaptation to training [45]: before (L0) and immediately after each training session (i.e., L1, L2, L3, L4, and L5) (Figure 1).

### 2.4. Blood Sampling and Isolation of Peripheral Blood Mononuclear Cells (PBMCs)

Before, 3 h and 24 h after the first training session (after 3 h and after 24 h), and 24 h after the last training session (after 5 d), fasted blood samples were drawn from the antecubital vein while subjects remained in reclined position (Figure 1). Blood sampled in EDTA tubes (BD Biosciences) was used for plasma collection by centrifugation of whole blood (2500 rpm × 10 min at 4°C) and for PBMC isolation. Human PBMCs were purified from whole blood by Ficoll gradient (Sigma-Aldrich, Milan, Italy), as already described [46]. Plasma and PBMC samples were aliquoted and stored at −80°C for further analyses.

### 2.5. Protein Extraction and Immunoblot Analysis

As previously reported, cells were lysed in RIPA buffer (150 mM NaCl, 50 mM tris-HCl pH 8, 1 mM EDTA, 1% NP40, 0.25% sodium deoxycholate, 0.1% SDS, water to volume) [47], supplemented with protease and phosphatase inhibitor cocktails (Sigma-Aldrich). Protein concentration was determined by a colorimetric assay using the BCA protein assay kit (Sigma-Aldrich). For the immunoblot analysis, an equal amount of proteins (20-30 *μ*g) was resolved in SDS-polyacrylamide (BIO-RAD) gels (10-12%) and transferred onto PVDF membranes (Amersham). Saturated membranes with 5% nonfat dry milk in PBS-Tween (0.01%) were incubated overnight with specific primary antibodies. The immune-reactive protein bands were detected by incubation with horseradish peroxidase-conjugated secondary goat anti-rabbit (Millipore) or goat anti-mouse (Sigma-Aldrich) antibodies. The western blot images were acquired on an ImageQuant LAS 4000 (GEHC) and quantified by ImageJ 1.50 h software (National Institutes of Health, USA, http://imagej.nih.gov/ij). The list of antibodies utilized is reported in Supplementary Table 1.

### 2.6. RNA Extraction and RT-qPCR Analysis

Total RNA was obtained from PBMCs by using the TRIzol (Invitrogen) reagent, according to the manufacturer's procedure. As previously described [48], RNA was digested with RNAse-free DNAse (Ambion). Real-time quantitative RT-qPCR was performed on a 7500 Real-Time PCR System (Applied Biosystems, Life Technologies). Each reaction mixture contained Power SYBR Green RNA-to Ct 1stepMaster mix (2x) (Life Technologies), specific primer sets, RT Enzyme Mix (125x) (Life Technologies) of RNA samples. All samples were run in triplicate. Values obtained for the target gene were compared with values of an internal control gene, Cyclophilin A. A threshold cycle (Ct) was observed in the exponential phase of amplification, and quantification of relative expression levels was performed with standard curves for target genes (*Δ*Ct) and the endogenous control (*ΔΔ*Ct). Geometric means were used to calculate the *ΔΔ*Ct (delta-delta Ct) values and expressed as 2^-*ΔΔ*Ct^. The value of each control sample was set at 1 and was used to calculate the fold change of target genes. Primers were designed using Primer 3 Plus (http://www.bioinformatics.nl/cgi-bin/primer3plus/primer3plus.cgi) and Primer-Blast using the reference and the alternative RefSeq accession numbers. The list of primers is reported in Supplementary Table 2.

### 2.7. Protein Carbonyl Content

The Protein Carbonyl Colorimetric Assay Kit (Cayman Chemical Company, USA) was performed in plasma samples using the manufacturer's procedure. The protocol is based on the reaction between 2,4-dinitrophenylhydrazine (DNPH) and protein carbonyls forming Schiff base. The concentration of C=O is expressed per total protein content (nmol/mg protein).

### 2.8. Multiplex Cytokine Assay

Plasma levels of IL8, IL6, IL10, IL17E, IL17F, IL21, IL22, and IL23 were assayed in subjects experiencing the physical activity program, preexercise (before) and 24 h after the last training session (after 5 d), using a magnetic bead-based multiplex assay (Milliplex Human Cytokine, Chemokine Assay, Millipore-Sigma, Merck) according to the manufacturer's recommendation and as previously described [49]. Data acquisition was performed by Bio-Plex 200 System™ (Bio-Rad Laboratories, Inc.), which uses Luminex fluorescent-bead-based technology (Luminex). Data analysis was performed by Bio-Plex Manager™ 6.0 software (Bio-Rad Laboratories, Inc.). Plasma samples were run in triplicate twice.

### 2.9. Statistical Analysis

The statistical analysis was conducted using GraphPad Prism software 8.0 (GraphPad software, San Diego, CA). The Kolmogorov-Smirnov or Shapiro-Wilk test was used to test the normality of quantitative variables. Normally distributed, continuous variables were analyzed using one-way ANOVA for repeated measures with Bonferroni correction as the post hoc test and Student's *t*-test. A correlation matrix was used to test the strength of any associations among variables. In all cases, *p* value ≤ 0.05 was considered significant.

## 3. Results

### 3.1. Baseline Characteristics of Subjects

The recruited male subjects show a VO_2max_ average of 41.8 ± 3.8 mL/kg/min). This fitness level falls within the range of 38 < VO_2max_ > 56 commonly seen in most of the physically active populations that regularly participate in nonelite sports/recreational activities [50, 51]. The anthropometric and fitness levels of the experimental group are shown in Table 1. All participants reported similar eating habits with regard to the percentage of macro- and micronutrients consumed daily (data not shown).

### 3.2. Blood Lactate and Heart Rate Analysis

**Figure 2:**
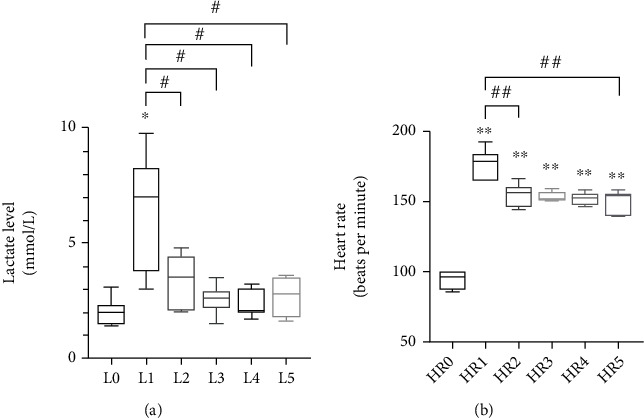
Blood lactate level (a) and heart rate (b) measured in subjects at different times before (L0) and immediately after (L1, L2, L3, L4, and L5) each training session. The values are presented as means ± SEM. ^∗^*p* < 0.05 and ^∗∗^*p* < 0.01 vs. before training period; ^#^*p* < 0.05; ^##^*p* < 0.01.

Results in Figure 2(a) show an increase of the blood lactate level immediately after the first exercise session (L1 *vs.* L0: 2.03 mmol/L ± 0.22*vs.*6.47 ± 0.95, *p* < 0.05), whereas its level remains at the baseline level at each time point after the following exercise sessions (L2-L5) without significant changes (*p* > 0.05).

As shown in Figure 2(b), the highest heart rate is recorded after the first exercise session (HR0 *vs.* HR1: 94.57 bpm ± 2.26*vs.*177.57 ± 9.78, *p* < 0.01), and compared with the baseline level, it remains higher immediately after the additional training sessions (HR0 vs. HR3, HR4, HR5: 94.57 bpm ± 2.26*vs.*154.00 ± 1.15, 151.43 ± 1.54, 149.14 ± 2.91, *p* < 0.01). Nevertheless, the values after the 2^nd^ to the 5^th^ training sessions (HR2-HR5) significantly decreased compared with the 1^st^ session (HR1 *vs.* HR2: 177.57 bpm ± 9.78 vs. 154.14 ± 7.88, *p* < 0.01).

### 3.3. Systemic Oxidative Damage Measured at Plasma Level and in PBMCs

**Figure 3:**
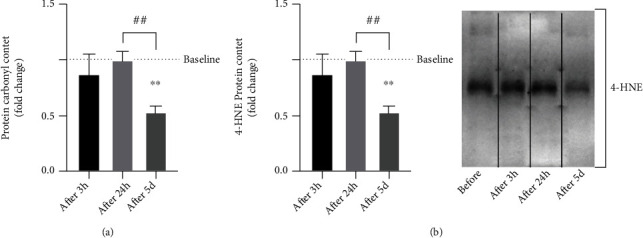
Analysis of plasma level of protein carbonyl content (a) and lipid peroxidation level (b) in PBMCs at baseline (before), after 3 hours (after 3 h) and 24 hours (after 24 h) following the first training session, and after 24 h (after 5 d) following the last training session in healthy subjects (*n* = 10). Bars in each histogram show the fold changes related to the baseline level measured before starting the training period. Data are presented as the means ± SEM. Statistical significance was determined using ANOVA with Bonferroni's post hoc analysis. ^∗^*p* < 0.05 and ^∗∗^*p* < 0.01 vs. baseline; ^##^*p* < 0.01.

No changes in plasma protein carbonylation or PBMCs 4-HNE are found after 3 h and 24 h from the first exercise session (*p* > 0.05) (Figures 3(a) and 3(b)).

The results show a significant reduction in plasma protein carbonylation content at the end of the training period (after 5 d) compared with the baseline levels (0.5 ± 0.05 fold change, *p* < 0.05) as well as its value measured after 24 h from the end of the first exercise session (*p* < 0.01) (Figure 3(a)).

A similar result is detected for lipid peroxidation, which demonstrates a decrease of HNE-protein-adducts at the end of the training period compared to the basal level (0.55 ± 0.10 fold change, *p* < 0.05) (Figure 3(b)).

### 3.4. Gene Expression and Protein Analysis of Antioxidants in PBMCs

**Figure 4:**
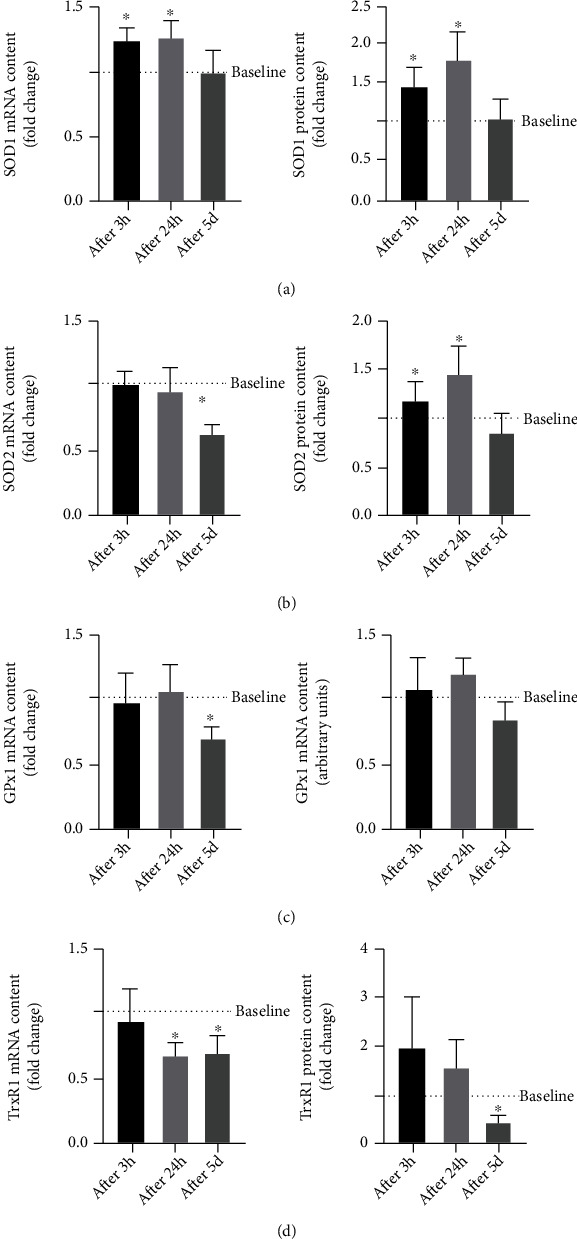
Analysis of proteins and mRNA levels of antioxidants SOD1 (a), SOD2 (b), GPx1 (c), and TrxR1 (d) at baseline (----), after 3 hours (after 3 h) and 24 hours (after 24 h) following the first training session, and after 24 h following the last training session (after 5 d) in healthy subjects (*n* = 10). Bars in each histogram show the fold changes related to the baseline level measured before starting the training period. Data are presented as the means ± SEM. Statistical significance was determined using ANOVA with Bonferroni's post hoc analysis. ^∗^*p* < 0.05 vs. baseline.

As shown in Figure 4, the analysis of several antioxidants reveals a prompt response in terms of gene expression of SOD1, which is significantly increased already after 3 h following the first exercise session (after 3 h, 1.26 ± 0.07 fold change; after 24 h, 1.28 ± 0.11, *p* < 0.05), returning at the basal level at the end of the training period (before *vs.* after 5 d, *p* > 0.05). The protein level of SOD1 is significantly increased only after 24 h of the first exercise section (after 24 h, 1.80 ± 0.34, *p* < 0.05). No changes in mRNA and proteins are observed after 5 d of training with respect to the baseline level (*p* > 0.05) (Figure 4(a)).

The analysis of SOD2 and GPx1 highlights a similar modulation with a significant decrease of their mRNA at the end of the training period (after 5 d, SOD2: 0.63 ± 0.06 fold change, *p* < 0.05; GPx1: 0.69 ± 0.07 fold change, *p* < 0.05), while no differences are observed for the protein levels (*p* > 0.05) (Figures 4(b) and 4(c)). No acute response is detected for both SOD2 and GPx1.

Interestingly, the mRNA level of TrxR1 is decreased both at 24 h following the first training session (after 24 h, 0.67 ± 0.09 fold change, *p* < 0.05) and at the end of exercise training protocol (after 5 d, 0.69 ± 0.12 fold change, *p* < 0.05) compared with the baseline level, whereas its protein level results are negatively modulated only at the last experimental point (after 5 d, 0.49 ± 0.11 fold change, *p* < 0.05) (Figure 4(d)).

No significant changes are observed at mRNA and protein levels of CAT (*p* > 0.05) at any experimental point (data not shown).

### 3.5. Modulation of Heat Shock Proteins at mRNA and Protein Levels in PBMCs

**Figure 5:**
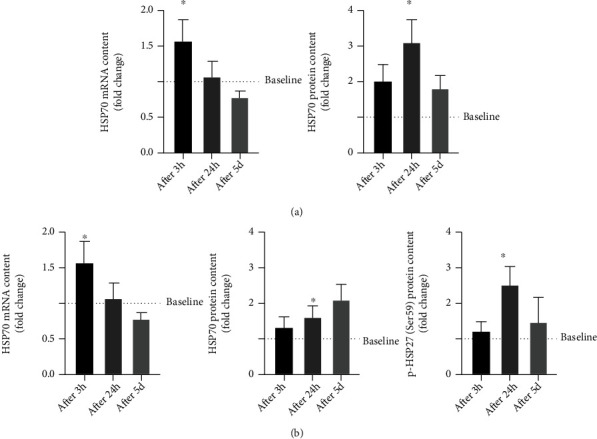
Analysis of proteins and mRNA levels of heat shock proteins HSP70 (a) and HSP27 (b) at baseline (----), after 3 hours (after 3 h) and 24 hours (after 24 h) following the first training session, and after 24 h following the last training session (after 5 d) in healthy subjects (*n* = 10). Bars in each histogram show the fold changes related to the baseline level measured before starting the training period. Data are presented as the means ± SEM. Statistical significance was determined using ANOVA with Bonferroni's post hoc analysis. ^∗^*p* < 0.05 vs. baseline.

The analysis of HSP modulation highlights a significant increase of mRNA HSP70 already after 3 h from the first exercise session (after 3 h, 1.59 ± 0.28 fold change, *p* < 0.05), returning to baseline levels 24 hours after exercise (after 24 h and after 5 d, *p* > 0.05). HSP70 protein content results upregulated at 24 h following the first exercise session (after 24 h, 3.40 ± 0.58 fold change, *p* < 0.05), whereas no differences are observed at the end of exercise training (after 5 d, *p* > 0.05) (Figure 5(a)).

Similarly, the HSP27 mRNA level is increased immediately at 3 h after the first exercise session (after 3 h, 1.49 ± 0.09 fold change, *p* < 0.05), while the protein level increases at 24 h following the first exercise session (after 24 h, 1.81 ± 0.20 fold change, *p* < 0.05). The phosphorylation level of HSP27 results significantly modulated only after 24 h from the first exercise session (after 24 h, 2.38 ± 0.95 fold change, *p* < 0.05) (Figure 5(b)).

It is not possible to detect appreciable levels of *α*B-crystallin in the PBMCs from all participants, neither at RNA nor protein level, questioning its actual expression in nonproliferating leukocytes (data not shown).

### 3.6. Cytokine Profile Analysis following the Exercise Training Period

**Figure 6:**
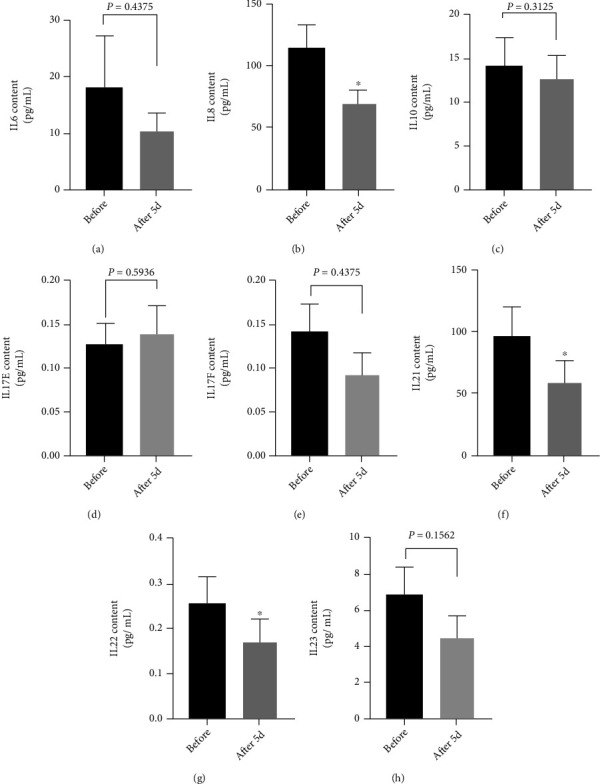
Plasma levels of (a) IL6, (b) IL8, (c) IL10, (d) IL17E, (e) IL17F, (f) IL21, (g) IL22, and (h) IL23 in healthy subjects (*n* = 10) experiencing physical activity program, preexercise (before) and 24 h after the last training session (after 5 d). Data are presented as the means ± SEM. Statistical significance was determined using Student's *t*-test analysis. ^∗^*p* < 0.05.

The level of IL6, IL10, IL17E, IL17F, and IL23 is not changed significantly in response to exercise training (*p* > 0.05) (Figure 6). On the contrary, the mean levels of IL8, IL21, and IL22 are significantly decreased after the 5-day training protocol as compared to baseline values: IL8 decreased from 114.67 ± 18.8 to 68.9 ± 10.89 pg/mL (*p* < 0.05) (Figure 6(b)), IL21 is decreased from 101.68 ± 23.56 pg/mL to 61.47 ± 18.79 pg/mL (*p* < 0.05) (Figure 6(f)), and IL22 is decreased from 0.26 ± 0.06 pg/mL to 0.17 ± 0.05 pg/mL (*p* < 0.05) (Figure 6(g)).

### 3.7. NF*κ*B Activation in PBMCs

**Figure 7:**
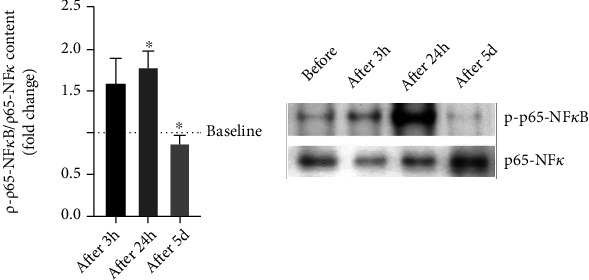
Western blot analysis of p-p65-NF*κ*B in PBMCs of subjects at baseline (----), after 3 hours (after 3 h) and 24 hours (after 24 h) following the first training session, and after 24 h following the last training session (after 5 d) in healthy subjects (*n* = 10). Bars in each histogram show the fold changes related to the baseline level measured before starting the training period. Data are presented as the means ± SEM. Statistical significance was determined using ANOVA with Bonferroni's post hoc analysis. ^∗^*p* < 0.05 vs. baseline.

As shown in Figure 7, the level of p-p65-NF*κ*B is positively modulated by acute exercise, showing a significant increase after 24 h from the first exercise session (after 24 h, 1.81 ± 0.17 fold change, *p* < 0.05), whereas at the end of exercise training, it is found below the basal level (after 5 d, 0.88 ± 0.09 fold change, *p* < 0.05).

### 3.8. Matrix Correlation Analysis

To verify the impact of exercise training in the correlation among molecules belonging to stress proteins, antioxidant/oxidative stress, and cytokine response, we considered the fold change in the protein level at the end of training with respect to the baseline level. We identified a positive correlation of HSP70 with catalase (HSP70 vs. CAT: *r* = 0.85; *p* = 0.033) and SOD1 (HSP70 vs. SOD1: *r* = 0.88; *p* = 0.021), while only a negative correlation was found between IL21 and catalase (IL21 vs. CAT: *r* = −0.95; *p* = 0.012) (Supplementary Figure 1).

## 4. Discussion

**Figure 8:**
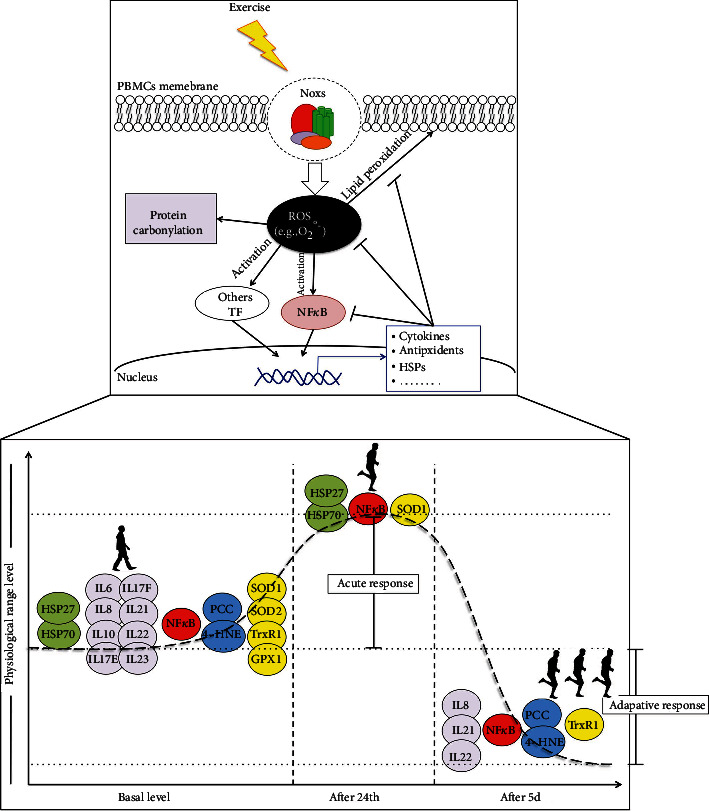
Proposed mechanism for the systemic response to moderate exercise in healthy adult subjects. Physiological increase of ROS concentration through exercise-induced NADPH oxidase is a potent intracellular stimulus for the transient expression/activation of molecules involved in antioxidant (GPX1, TrxR1, SOD1, and SOD2) and stress (HSP70 and HSP27) response, as well as in pro- and anti-inflammatory processes (IL8, IL10, IL17E, IL17F, IL21, IL22, and IL23). Following a single bout of moderate endurance exercise in untrained subjects, physiological levels of ROSs induce the transient upregulation of SOD1, HSP70, and HSP27, possibly involving NF*κ*B as an upstream mediator. Differently, 5 days of exercise training appears to be more reflective of a longer-term training adaptation reducing the content of TrxR1, IL8, IL21, and IL22 and damaged macromolecules, as well as the activation of NF*κ*B, indicative of positive effects of exercise training on the redox balance and the immunoresponse. ROS: reactive oxygen species; HSPs: heat shock proteins; PCC: protein carbonyl content; GPx1: glutathione peroxidase 1; NF*κ*B: nuclear factor kappa B; SOD1: copper-zinc superoxide dismutase; SOD2: manganese superoxide dismutase; TrxR1: thioredoxin reductase 1; TF: transcription factors; NOXs: NADPH oxidases; 4HNE: 4-hydroxynonenal.

To date, no studies conducted in humans have examined the effect of short-term exercise programs on circulating markers of oxidative stress, inflammation, and stress response proteins. For the first time, here, we show that five sessions of a regular and moderate exercise program, administered daily, are able to produce an improvement in immune health in untrained young adult subjects. In particular, we verified an acute response after the first exercise session that activated the antioxidant response as evidenced by the increase in PBMC SOD1 expression, as well as the upregulation/activation of specific stress response proteins such as HSP70 and HSP27. Interestingly, at the end of the 5-day training protocol, we observed a significant decrease of the basal level of oxidized proteins and lipids, as well as the reduction in PBMC antioxidants and circulating proinflammatory cytokines, which may suggest a very early beneficial adaptation phase to physical exercise (Figure 8).

When comparing studies looking at the inflammatory and stress protein response to exercise, it is important to compare subjects at the same physical level and with homogeneous biological and anthropometric parameters, such as age and BMI. Moreover, results from the blood lactate (L) and heart rate (HR) analyses, considered reliable markers of physiological adaptation to exercise training, show a homogeneous response within subjects. As expected, L and HR values increased immediately after each bout of exercise, although the increment resulted significantly lower already after the second exercise session. Although the homogeneity and limited number of subjects could impact the actual consistency of these parameters, their reduction after training is considered a good indicator of adaptation [45, 52], suggesting the potential effect of our exercise protocol in improving aerobic training capacity in the subject group.

The reduction in protein carbonylation and lipid peroxidation observed at the end of the training period, paralleled to the downregulation of SOD2, Gpx1, and TrxR1, are important indicators of a better redox status, which can represent an efficient combination between oxidant and antioxidants [53, 54]. Indeed, the type and timing of modifications in the expression of antioxidant enzymes (SODs, TrxRs, and GPxs) may explain the reduction of oxidized molecules in both PBMCs and plasma determined in untrained subjects after the physical training program. In 2011, Jenkins et al. demonstrated that in untrained healthy males, whose age, BMI, and aerobic capacity were comparable to that of our experimental group, the baseline levels of reactive nitrogen and oxidative species in PBMCs were higher when compared to matched trained individuals because of an increased NADPH oxidase activity in the untrained group [55]. Moreover, we and other groups demonstrated that the baseline levels of SOD1 were lower in untrained than in trained matched subjects [30, 55]. Thus, even in healthy young individuals, the absence of regular exercise training correlates with a higher prooxidant environment in immune cells [55–58].

Our results also confirmed that unaccustomed moderate training did not increase oxidative damage [54]. On the contrary, it represents a clear example of oxidative eustress stimulus [59], able to activate a prompt cellular response, as demonstrated by the transient upregulation of SOD1, HSP70, and HSP27, at both mRNA and protein levels, possibly involving NF*κ*B as an upstream or downstream mediator [60, 61]. We postulate that, in our protocol, an increase in cytoplasmic H_2_O_2_, from NADPH oxidase or mitochondrial activities, might represent the main driver for the acute response, largely involving the cytoplasmic superoxide dismutase and chaperone network [62].

It has been already demonstrated that regular participation in physical activity modulates in PBMCs the expression of molecules involved in the antioxidant response such as SOD2, TrxR1, OXR1, CAT, GPx, and UCP3 [8, 10, 12, 27, 63]. Similar to the aforementioned results, and matching both the subject's characteristics recruited and the type of exercise protocol, we found a return to the basal level of SOD1 expression and a significant reduction at mRNA and/or protein levels for SOD2, GPx1, and TrxR1 at the end of the short-term aerobic training, correlated with the lower amount of lipid peroxidation in PBMCs and plasma protein carbonylation.

The results obtained so far confirm the crucial role of ROS signalling in the physical exercise response and as a moderate, short-term exercise training significantly ameliorating the redox homeostasis at the systemic levels, thereby causing a better-adapted antioxidant asset in active people [64]. In particular, it is known that an acute bout of exercise at sufficient intensity stimulates expression/activities of antioxidant enzymes in the first 3-24 hours postexercise [63, 65, 66]. This could be considered a defensive mechanism of the cell under oxidative stress. However, a repeated exercise may induce a transient reduction of specific antioxidants as adaptive response to exercise training and thereby index of a better redox balance [8, 10, 12, 27, 63, 67].

It has been demonstrated that after acute exercise, a significantly greater percentage of leukocytes express HSP70, HSP90, HSP60, and HSP27 depending upon exercise intensity [26, 68–70]. Differently, there are no studies investigating the modulation of HSPs in leukocytes of adult subjects after a period of endurance exercise training. However, it is known that trained subjects show at rest a downregulation of HSP positive cells, which may reflect adaptation mechanisms to regular endurance training [69, 71]. Similarly to the aforementioned results, we found that both HSP70 and HSP27 were upregulated in the PBMCs after acute exercise, whereas at the end of the training period their expression was returned to basal levels. These results confirm the prompt response of HSPs to exercise-induced stimuli and highlight the need for a longer training period for their adaptation. Protection and/or tolerance against exercise-induced oxidative, heat, cytokine, and inflammatory stress in leukocytes are provided by the modulation of other stress response proteins, including the HSPs [72, 73]. In different tissues, HSPs play a role in protein translocation, stabilization, assembly, and degradation processes, functions that could be important in leukocytes activated by physical exercise [30, 69, 74–77]. It is also known that HSPs are able to function as powerful cytokines by binding to Toll-like receptors 2 and 4 (TLR2 and TLR4) [78, 79]. TLRs are the critical sensors for the recognition of microorganisms whose expression patterns are closely related to the immunologic function of the cells [80].

Regular participation in physical exercise has a positive effect on the immune system [14]. Although it has been demonstrated that physiological response to acute and long-term adaptations of immunity to exercise is dependent on exercise characteristics (i.e., type, intensity, frequency, and duration), to date exercise training can be considered a kind of “immunotherapy” capable of promoting an anti-inflammatory environment or attenuating the acute response to exercise, possibly reducing the risk of developing inflammatory-related diseases [7, 81, 82]. Moreover, the available scholarly literature seems to suggest its positive effects on immune responses and outcomes to viral infections [33, 83].

To investigate in detail the relationships between short-lasting exercise protocol and systemic inflammatory markers, we analyzed the expression of numerous cytokines. Similar to previous researches [84–86], we found that a healthy amount of regular exercise reduces levels of inflammatory markers. Particularly, exercise training induced a tendency to decrease all cytokines, with someone reaching significance, including the proinflammatory IL8 and both IL21 and IL22 produced by Th17 cells, which play a critical role in the pathogenesis of autoimmune diseases [87].

Taken together, these data show that only 5 days of exercise training appear to be more reflective of a longer-term training adaptation and may be indicative of an effect of exercise training in reducing the risk of developing inflammatory-derived and autoimmune diseases.

With respect to the possible interconnection between the inflammatory, antioxidant, and HSP responses, nuclear factor (NF) *κ*B is known to play a critical role in mediating immune and inflammatory/oxidative responses and apoptosis [88]. NF*κ*B signalling pathways may be triggered by several stimuli such as ROS [54, 89], HSPs [79], HO-1 [56], and cytokines [90] induced by regular physical activity. As already demonstrated by Ji and colleagues [91], we found that p-p65-NF*κ*B tends to increase after 3 h from the end of the first training session, reaching significance after 24 h. When this parameter was analyzed at the end of the training period, the activity of this protein was observed significantly reduced even compared with the basal level. Since the NF*κ*B signalling pathway is induced in a redox-sensitive manner, the hypothesis that the activation of this protein is partly determined by the alteration of the redox state induced by exercise is convincing. Indeed, a reduction of systemic oxidative stress markers observed following the training period was parallel to the reduction of NF*κ*B activity and antioxidant content [54]. Moreover, an increasing number of studies have shown that Hsp70 may be involved in the regulation of NF*κ*B activity, protecting from the inflammatory response by preventing either NF*κ*B activation directly [92] or via TRAF6, an essential activator of the NF*κ*B pathway [93]. Another important protein regulating the fine balance of cellular redox status and responses to stress and inflammation in lymphocytes is the nuclear factor erythroid 2-related factor 2 (Nrf2) [94]. Although we did not measure the protein levels of Nrf2, as previously stated [95], it could speculate a further modulation of this transcription factor in response to physical exercise.

Finally, the correlation analysis among different categories of molecules (i.e., antioxidants, stress response, and inflammatory) performed at the end of the training period highlights a parallel increase of HSP70 expression, SOD1, and CAT, as well as an opposite trend for CAT and IL21. Although the causal link between these molecules remains purely speculative in this study, it is known that an acute prooxidant stimulus (acute exercise) can induce the expression of these biomolecules (i.e., HSP70, SOD1, and CAT) to maintain the cellular homeostasis, while in a condition of “exercise adaptation,” where the ROS production is better balanced by the various cellular systems, their expression can be reduced. The inverse correlation between IL21 and CAT could explain the further role of this interleukin in immune cells, where it could trigger an immunometabolic axis including improved mitochondrial fitness and cellular redox status [96]. Taken together, these results point out a conceivable relationship among antioxidants, HSP induction, and cytokines in immunocompetent cells. As already demonstrated by others [69, 97], these correlations help us to complete the complex puzzle where moderate exercise induces activation of immunocompetent cells, followed by an increase of ROS and cytokines, resulting in HSP induction (Supplementary Figure 1).

Some limitations of this study have to be considered. Our results were obtained on male subjects; this could make the results not generalizable to females; in the same instances, the small sample size may have reduced the number of statistical differences; lack of experimental point 48 h after the last exercise session and mechanistic measurements could better clarify the specific effect of exercise training on the analyzed parameters.

Moreover, although our results show trends in the direction of specific biomarker changes over time, we cannot attribute the absolute biomarker changes entirely to our intervention because the absence of a “nonexercised” control group does not allow the exclusion of additional factors.

## 5. Conclusion

To our knowledge, this is the first study to reveal an early adaptive response of immune cells after a successful 5 days of moderate exercise training in untrained healthy subjects with a medium fitness level.

The exact function of the differential regulation of all molecules analyzed in response to exercise training remains to be further investigated. However, it is intriguing to speculate that this type of physical activity conducted in frail people, such as the elderly, could lead to a reduction in chronic inflammation and oxidative stress, which result to be physiologically increased in PBMCs with age and linked to immunosenescence [98–100]. As demonstrated with other long-duration moderate aerobic exercises [33, 83], our results could also suggest in the long term an improvement of influenza risk and increased rates of vaccine efficacy.

To increase the reliability and applicability of the results, further studies need to investigate the effect of this exercise protocol on a greater number of subjects, possibly extending the analysis on female subjects and on a fragile population such as the elderly. Due to the dependence of changes in the activity of antioxidant enzymes after physical exercise, future research should also include this analysis in PBMCs and at the plasma/serum level, as well as a detailed analysis of blood lymphocyte phenotypic characteristics induced at the end of our exercise training. Moreover, they remain strongly recommended researches, preferably long-term standardized mechanistic studies, to understand the impact of the observed change in gene and protein expression in leukocytes and other organs, as well as to understand the complex interaction between white blood cells and muscle inflammatory response.

## Figures and Tables

**Table 1:** Subject characteristics.

	Untrained (*n* = 10)
Age (yrs)	26.6 ± 3.1
Weight (kg)	71.5 ± 12.5
BMI (kg/m^2^)	23.7 ± 3.6
HR (bpm)	94.9 ± 4.7
VO_2max_ (mL/kg/min)	41.8 ± 3.8
Work index (score)	2.3 ± 0.6
Sports index (score)	3.2 ± 2.1
Leisure index (score)	2.8 ± 0.5

Values are means ± SD. BMI: body mass index; HR: heart rate; VO_2max_: maximal aerobic capacity.

## Data Availability

Data available on request.

## Supplementary Materials

Supplementary Materials Supplementary Figure 1: heat map representation of the correlation matrix among fold changes (after 5 d/before) of molecules belonging to stress proteins, antioxidant/oxidative stress, and inflammatory response. The *r* value of the correlation is indicated in each cell of the matrix. Supplementary Table 1: list of primary antibodies utilized. Supplementary Table 2: sequences of the oligonucleotides used for RT-qPCR.
