# Supplementary material for: Systemic Response of Antioxidants, Heat Shock Proteins, and Inflammatory Biomarkers to Short-Lasting Exercise Training in Healthy Male Subjects
Source: Oxid Med Cell Longev. 2021 Nov 22;2021:1938492. doi: 10.1155/2021/1938492 (PMC8629640; doi:10.1155/2021/1938492)

Supplementary Table 1. List of primary antibodies utilized.

Antigen	Product number	Dilution	Manufacturer
**β-actin**	sc-47778	1:3000	Santa Cruz
**GAPDH**	MAB 374	1:3000	Millipore
**αBcrystallin (HSPB5)**	ADI-SPA-222	1:2000	Enzo Life Sciences
**phospho-αBcrystallin (S59) (p-HSPB5)**	ADI-SPA-227	1:1000	Enzo Life Sciences
**4-hydroxynonenal**	ab46545	1:1000	Abcam
**HSP70 (HSP1A1)**	ADI-SPA-810	1:2000	Enzo Life Sciences
**HSP27 (HSPB1)**	sc-1048	1:1000	Santa Cruz
**phospho-HSP27 (p-HSPB1)**	#2401	1:1000	Cell Signaling
**ZnCuSOD (SOD1)**	sc-101523	1:500	Santa Cruz
**MnSOD (SOD2)**	SOD-110	1:1000	Stressgen
**CATALASE**	sc-271803	1:1000	Santa Cruz
**GPx1**	bs-3882R	1:1000	Bioss Antibodies
**TrxR1**	sc-20147	1: 1000	Santa Cruz
**p-NFκB p65**	sc-166748	1: 1000	Santa Cruz
**NFκB**	sc-372	1: 1000	Santa Cruz

Supplementary Table 2. Sequences of the oligonucleotides used for RT-qPCR.

Target Gene	FW sequence	Rev sequence
SOD1	5’- AAAGATGGTGTGGCCGATGT - 3’	5’- GCCAATGATGCAATGGTCTCC - 3’
SOD2	5’- CCCTGGAACCTCACATCAAC - 3’	5’- GGTGACGTTCAGGTTGTTCA - 3’
TrxR1	5’-ACAAGCCCTGCAAGACTCTC-3’	5’-AGCCCCAATTCAAAGAGCCA-3’
GPx1	5’-ACGATGTTGCCTGGAACTTT-3’	5’-TCGATGTCAATGGTCTGGAA-3’
HSP70	5’- AGGGGCCTTTCCAAGATTGC - 3’	5’- GCAAACACAGGAAATTGAGAACT - 3’
HSP27	5’- GCAGGACGAACATGGCTACAT - 3’	5’- TGGAGGCAGCGTGTATTTCC - 3’
GAPDH	5’- TGGTCACCAGGGCTGCTT - 3’	5’ - CATGTAGTTGAGGTCAATGAAGG - 3’
Cyclo A	5’ - GTCAACCCCACCGTGTTCTT - 3’	5’- CTGCTGTCTTTGGGACCTTGT - 3’

Supplementary figure 1

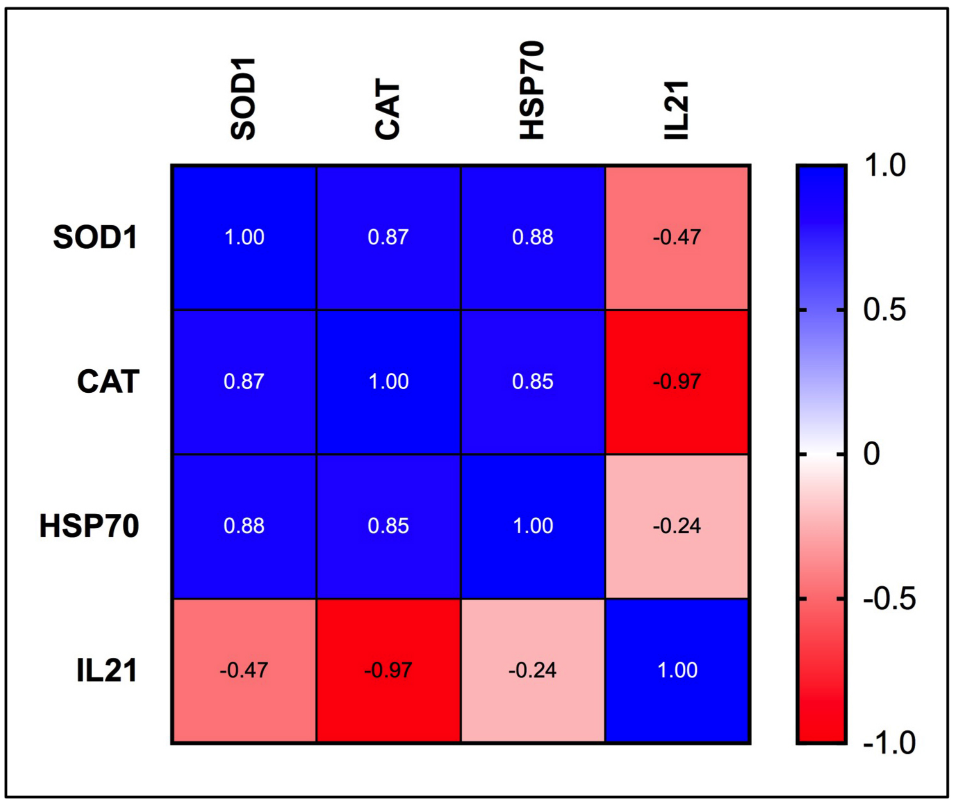

Supplement: Supplementary Materials — Supplementary Figure 1: heat map representation of the correlation matrix among fold changes (after 5 d/before) of molecules belonging to stress proteins, antioxidant/oxidative stress, and inflammatory response. The r value of the correlation is indicated in each cell of the matrix. Supplementary Table 1: list of primary antibodies utilized. Supplementary Table 2: sequences of the oligonucleotides used for RT-qPCR. [file 1938492.f1.zip › updated Supplementary Materials.pdf]
