# Supplementary figures and images for: Systemic Response of Antioxidants, Heat Shock Proteins, and Inflammatory Biomarkers to Short-Lasting Exercise Training in Healthy Male Subjects
Source: Oxid Med Cell Longev. 2021 Nov 22;2021:1938492. doi: 10.1155/2021/1938492 (PMC8629640; doi:10.1155/2021/1938492)

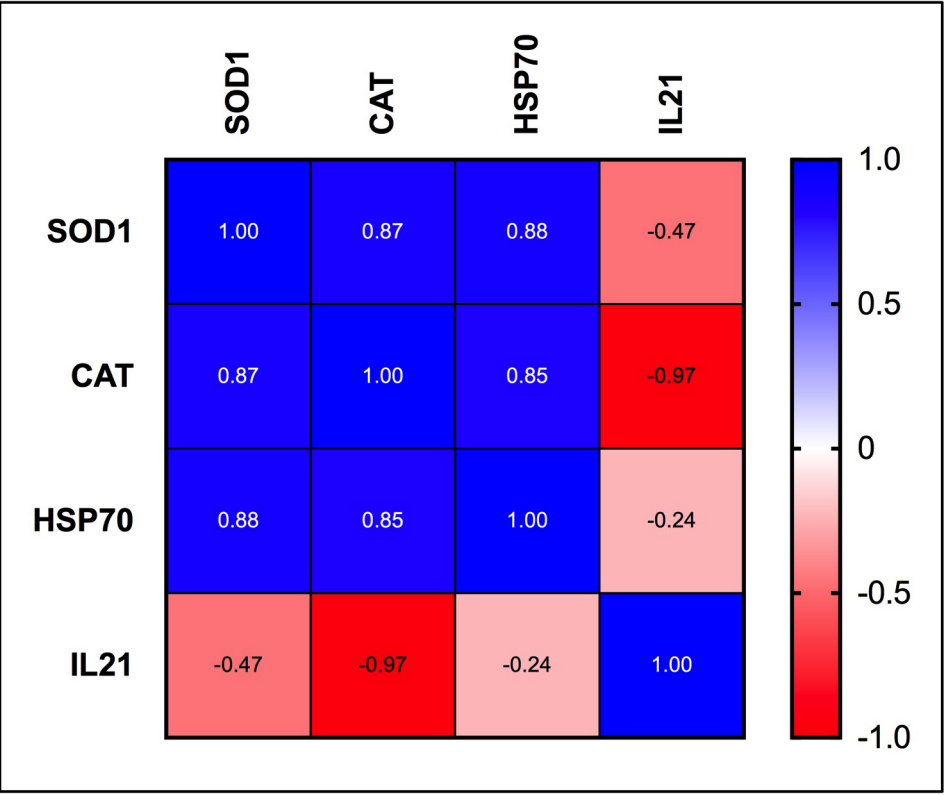

Supplement: Supplementary Materials — Supplementary Figure 1: heat map representation of the correlation matrix among fold changes (after 5 d/before) of molecules belonging to stress proteins, antioxidant/oxidative stress, and inflammatory response. The r value of the correlation is indicated in each cell of the matrix. Supplementary Table 1: list of primary antibodies utilized. Supplementary Table 2: sequences of the oligonucleotides used for RT-qPCR. [file 1938492.f1.zip › Supplementary Figure 1.pdf]
